# An exploration of differences between Japan and two European countries in the self-reporting and valuation of pain and discomfort on the EQ-5D

**DOI:** 10.1007/s11136-017-1541-5

**Published:** 2017-03-25

**Authors:** Yan Feng, Mike Herdman, Floortje van Nooten, Charles Cleeland, David Parkin, Shunya Ikeda, Ataru Igarashi, Nancy J. Devlin

**Affiliations:** 1 Office of Health Economics, London, UK; 2 Formerly with Astellas Pharma BV, Leiden, The Netherlands; 3 MD Anderson Cancer Center, Houston, TX USA; 4 International University of Health and Welfare, Otawara, Japan; 5 University of Tokyo, Tokyo, Japan

**Keywords:** Patient-reported outcomes, EQ-5D, Self-reported pain/discomfort, Health state valuation, Between-country comparison

## Abstract

**Purpose:**

To investigate the systematic differences in the self-reporting and valuation of overall health and, in particular, pain/discomfort between three countries (England/UK, Japan, and Spain) on the EQ-5D.

**Methods:**

Existing datasets were used to explore differences in responses on the EQ-5D descriptive system between Japan (3L and 5L), the UK (3L), England (5L), and Spain (5L), particularly on the dimension of pain/discomfort. The role of different EQ dimensions in determining self-reported overall health scores for the EuroQol visual analog scale (EQ-VAS) was investigated using ordinary least squares regression. Time trade-off (TTO) results from Japanese and UK respondents for the EQ-5D-3L as well as Japanese and English respondents for the EQ-5D-5L were compared using *t* tests.

**Results:**

For the EQ-5D-3L, a higher percentage of respondents in Japan than in the UK reported ‘no pain/discomfort’ (81.6 vs 67.0%, respectively); for the EQ-5D-5L, the proportions were 79.2% in Spain, 73.2% in Japan, and 63–64% in England, after adjusting for age differences in samples. The ‘pain/discomfort’ dimension had the largest impact on respondents’ self-reported EQ-VAS only for EQ-5D-3L in Japan. Using the EQ-5D-3L, Japanese respondents were considerably less willing to trade off time to avoid pain/discomfort than the UK respondents; for example, moving from health state, 11121 (some problems with pain/discomfort) to 11131 (extreme pain/discomfort) represented a decrement of 0.65 on the observed TTO value in the UK compared with 0.15 in Japan. Using the EQ-5D-5L, Japanese respondents were also less willing to trade off time to avoid pain/discomfort than respondents in England; however, the difference in values was much smaller than that observed using EQ-5D-3L data.

**Conclusions:**

This study provides evidence of between-country differences in the self-reporting and valuation of health, including pain/discomfort, when using EQ-5D in general population samples. The results suggest a need for caution when comparing or aggregating EQ-5D self-reported data in multi-country studies.

## Introduction

Patient-reported outcomes (PROs) are widely used as outcome measures in clinical trials of pain treatments. Indeed, given that pain can *only* be measured subjectively, studies of pain are entirely reliant on self-reporting [1]. The assessment of pain is incorporated in different ways in PROs, ranging from generic measures of health-related quality of life, such as EQ-5D [2] in which pain/discomfort is only one of five dimensions measured, to tools that are specific to the assessment of pain, such as the Brief Pain Inventory [3]. One important consideration when using such measures in multi-country studies is that factors such as race, ethnicity, language, and culture can potentially affect responses to PRO instruments [4–6].

With regard to pain reporting, there is evidence that this can vary quite widely across countries; for example, the results of a survey published in 2014 showed that pain reporting and treatment rates were lower in China (6.2% and 28.3%, respectively) and Japan (4.4% and 26.3%, respectively) than in the other countries involved (≥14.3% and 35.8%, respectively) [7]. Substantial variations between countries in the rate of pain reporting have also been reported in primary care [8] and among patients with cancer [9]. Within Europe, studies have also shown variation in pain reporting; using the pain/discomfort dimension of the EQ-5D, rates of respondents declaring no pain/discomfort varied from 65% in France to 79.5% in Spain [10]. At least some of the difference between countries may depend on cultural differences in pain response rather than on differences in objective levels of pain. For example, in experimental studies, Japanese subjects provided lower pain ratings for equivalent ‘objective’ levels of pain than European subjects [11], whereas other studies have shown that Euro-Americans consider seeking pain relief more acceptable than Japanese respondents [12]. However, evidence from Uki et al. suggests that Japanese patients with cancer reported high levels of pain with inadequate pain management [13].

The EQ-5D is one of the most widely used generic preference-based measures of health status. It has been translated into numerous languages and is available in an increasing number of country-specific utility-based value sets. Nevertheless, little attention has been paid to whether the effects described earlier in relation to pain affect the self-rating and valuation of pain/discomfort on EQ-5D and whether such data can be compared and aggregated across countries. In one of the few such analyses performed, Tsuchiya et al. compared the results of the UK and Japanese EQ-5D-3L valuation data and noted that the two datasets were positively correlated [14]. Furthermore, Japanese time trade-off (TTO) values were consistently higher than those from the UK, except for mild states.

The availability of self-reported and valuation EQ-5D-3L data from Japan and the UK together with self-reported and valuation data from England and Japan for the latest version of the instrument, the EQ-5D-5L, makes it possible to explore these questions in more depth. Self-reported data were also available for the EQ-5D-5L from Spain. The primary aim of this study was to investigate whether available EQ-5D data provide evidence of systematic differences in the way respondents in Japan self-report and value health using EQ-5D compared with respondents from England/UK and Spain, with a particular focus on the pain/discomfort dimension.

## Methods

### The EQ-5D

The EQ-5D consists of a descriptive system and a visual analog scale (EQ-VAS). Respondents rate their health on the EQ-5D descriptive system and assess their overall health on the EQ-VAS.

There are two versions of the instrument for use in the adult population, EQ-5D-3L and EQ-5D-5L. Both measure health using a descriptive system with five dimensions (mobility, self-care, usual activities, pain/discomfort, and anxiety/depression). The original version of EQ-5D (EQ-5D-3L) uses three levels of severity (no, some, extreme problems or unable to) in each dimension. To increase the instrument’s sensitivity, a new version of the instrument (EQ-5D-5L) was developed with five levels of severity (no, slight, moderate, severe, extreme problems or unable to) [15]. Self-ratings on the descriptive system are summarized as a five-number ‘code’ where each number reflects the severity level on the individual dimensions. Each ‘code’ represents a unique health state, with the state 11111 representing full health. There are 243 EQ-5D-3L health states (243 = 3^5^) and 3125 EQ-5D-5L health states (3125 = 5^5^). By selecting one level of severity in each dimension on either version of the EQ-5D, respondents assign themselves one out of all possible health states as a description (known as a ‘profile’) of their own health.

The EQ-VAS consists of a vertical scale with anchor points of 0 (worst possible health) and 100 (best possible health). The respondent marks a point on the scale to show how they perceive their overall health.

In addition to providing these two types of self-reported data, the EQ-5D is frequently used in conjunction with health state valuation techniques such as TTO or discrete choice experiments to generate preference-based societal weights for each of the individual health states generated by the descriptive system [16]. It is recommended that weights be obtained for individual countries, as values assigned to health states might differ between countries for cultural or other reasons [17].

### Data

Valuation and self-reported health data obtained from the EQ-5D-3L valuation surveys in the UK and Japan as well as EQ-5D-5L valuation surveys in England and Japan were used [14, 18–20]. At the time the present study was carried out, self-reported health data on EQ-5D-5L were also available for Spain [21]. In all cases, the samples included were intended to be representative of the general population of the country.

Socio-demographic and health-related characteristics available in each of the five datasets included age, gender, and respondents’ experience of serious illness (in themselves, a family member, or others). The same background characteristics were recorded in each of the five datasets.

The Japanese EQ-5D-3L valuation study was a quasi-replication of the UK valuation study [14]. Each respondent valued the same set of the 17 health states, which were a subset of the 42 health states in the UK study [18]. The EQ-5D-5L valuation data used in the present analysis were collected during valuation studies carried out in Japan, England, and Spain using the EuroQol valuation technology (EQ-VT) software, which was developed specifically for the EQ-5D-5L value set studies [16]. An identical methodology based on computer-assisted personal interviews (CAPI) was used in all countries. Each respondent was asked to provide TTO values for a block of 10 health states out of the 86 health states selected for direct valuation. All interviewers received training on administration of the CAPI and EQ-VT. Only the observed TTO values (rather than the value sets modeled from those data) were used in this study.

A summary of the characteristics of each survey is provided in Table 1.

**Table 1:** Characteristics of the five survey datasets

Survey places	EuroQol version	Self-rating of EQ-5D profile and EQ-VAS	Valuation data	No. of health states in the TTO	Background variables^a^	Year of survey	No. of respondents	Exclusion criteria for the valuation study	No. of respondents in the valuation data set^b^	Software^c^	Health state valuation method
Japan	EQ-5D-3L	Available^d^	Available	17	Available	1998	621	Respondents completely missing TTO data; valued 1 or 2 states only; giving all states the same value; valued all states worse than dead	543	STATA	Conventional TTO
UK	EQ-5D-3L	Available	Available	42	Available	1993	3395	Missing in valuation data	2997	LIMDEP	Conventional TTO
Japan	EQ-5D-5L	Available	Available	86	Available	2014	1098	Three of the investigators (involving 72 respondents) did not follow the survey manual procedures	1026	STATA, SAS, and R	Lead time TTO
England	EQ-5D-5L	Available	Available	86	Available	2012–2013	996	Respondents who gave the same TTO value for all health states; giving health state 55555 a value no lower than the value for the mildest health state in their block	912	R and WinBugs	Lead time TTO and DCE
Spain	EQ-5D-5L	Available	N/A	86	Available	2012	1000	N/A	N/A	N/A	N/A

*N*/*A* not available, *TTO* time trade-off, *DCE* discrete choice experiments

^a^The background variables include age, gender, experience with serious illness by respondents themselves, their families, and others

^b^Represents the number of respondents after applying the exclusion criteria. For the Japanese EQ-5D-3L dataset, this study only has access to the sample that is included in the valuation study (*N* = 543)

^c^The software used for the original analysis in the valuation studies

^d^‘Available’ and ‘N/A’ refer to the availability of data for this study

### Statistical analysis

There were five parts to the statistical analysis. First, respondents’ socio-demographic and health-related characteristics were compared across the five datasets. Second, self-reported data on the descriptive systems of EQ-5D-3L and EQ-5D-5L were compared to investigate whether there were systematic differences between countries that could not be explained by differences in sample characteristics on age or gender, particularly in regard to reporting of pain/discomfort. Third, the impact of the five dimensions of the descriptive system on EQ-VAS scores was analyzed to determine, in particular, the contribution of the pain/discomfort dimension to VAS scoring. Fourth, valuation data were used to explore whether respondents in Japan and the UK for the EQ-5D-3L valuation studies and respondents in Japan and England for the EQ-5D-5L valuation studies have different stated preferences in terms of their willingness to trade off time in TTO tasks, particularly in relation to health states involving pain/discomfort. Fifth, the linked self-reported and valuation datasets for the EQ-5D-3L and EQ-5D-5L valuation studies in the UK/England and Japan were analyzed to investigate whether there was any relationship between respondents’ self-reported pain/discomfort and the TTO values they assigned to the hypothetical health states.

The socio-demographic and health-related characteristics of the different samples were compared using *t* tests for age, and chi-squared tests for gender and the proportions of respondents who reported having experienced serious illness in themselves, a family member, or others.

Respondents’ self-reported EQ-5D data were analyzed by comparing the distribution of EQ-5D profile data by country and instrument version. The ceiling effect (measured by the proportion of respondents reporting the best possible health for EQ-5D), the number of EQ-5D profiles used, and the distribution of responses by dimension were also calculated and compared across countries and EQ-5D versions. Adjustments for age and gender were made when comparing distributions on the descriptive system across countries. Using England as an example, to adjust for age difference, five age ranges were used (≤30, 31–45, 46–60, 61–75, and >75 years). For each age range, the proportion of respondents in England who reported full health was calculated followed by the weighted average of the five proportions. The weight for each age band is the proportion of respondents in that age band in Spain or Japan. Adjustments for any differences in gender distributions were made in the same way.

The extent to which responses on the five dimensions of the descriptive system explained self-reported overall health on the EQ-VAS was examined using ordinary least squares (OLS) regression methods and the results were compared between countries for both the 3L and 5L. Data on the five dimensions of the EQ-5D were recorded as continuous variables (1 for level 1, 2 for level 2, 3 for level 3, 4 for level 4, and 5 for level 5). To show the model’s goodness of fit, adjusted R-squared and results from residual analysis were reported after each regression.

To explore whether there were differences in TTO values in the valuation studies between respondents, in particular for pain/discomfort, EQ-5D-3L and EQ-5D-5L valuation data from the UK/England and Japan were analyzed. Respondents in the UK and Japan in the EQ-5D-3L studies yielded observed values for 42 health states in the UK and 17 health states in Japan. The EQ-5D-5L studies in Japan and England yielded observed values for 86 health states. TTO values in each version of the EQ-5D for those health states were compared between the two countries using *t* tests.

Among the 17 hypothetical health states in the EQ-5D-3L valuation data, there were three pairs of health states that *only* differed on the pain/discomfort dimension. Among the 86 hypothetical health states in the EQ-5D-5L valuation data, there were seven pairs of health states that *only* differed on the pain/discomfort dimension. For each version of the EQ-5D valuation studies, the difference in mean TTO values was compared between respondents by country (for those pairs that only differed in the pain/discomfort dimension) to gain insight into differences in how respondents in Japan and the UK/England value EQ-5D health states with respect to the pain/discomfort dimension.

Finally, respondents’ self-reported and valuation data were linked using respondents’ ID for the EQ-5D-3L and EQ-5D-5L data from Japan and the UK/England. Using both the self-reported and valuation data, the effect of self-reported pain/discomfort in explaining the TTO values was explored using the OLS regression analyses to model the TTO values for the five dimensions of the EQ-5D profile and self-reported pain/discomfort. All analyses were performed using STATA/MP 13.

## Results

There was no statistically significant difference in mean age between respondents in the UK and Japan in the EQ-5D-3L data (*P* = 0.753), although the UK sample had a higher proportion of females and respondents who had experienced serious illness in themselves or in a family member, and a lower proportion of respondents who had taken care of others with a serious illness (*P* < 0.05). There was no statistically significant difference in mean age between respondents in Japan and Spain in the EQ-5D-5L datasets (*P* = 0.119); however, respondents in England were older than respondents in Japan and Spain (*P* < 0.05). The English sample also reported the highest proportion of females and respondents who had experienced serious illness themselves, in a family member, or who had taken care of others with a serious illness (*P* < 0.05) (Table 2).

**Table 2:** Socio-demographic and health-related characteristics of respondents in Japan and the UK for the EQ-5D-3L; Japan, England, and Spain for the EQ-5D-5L

Respondent characteristics	EQ-5D-3L			EQ-5D-5L			
	Japan (*n* = 543)	UK (*n* = 3395)	*P* value	Japan (*n* = 1026)	England (*n* = 996)	Spain (*n* = 1000)	*P* value
Age, years, mean (SD)	48.1 (15.3)	47.9 (18.4)	0.753	44.9 (14.9)	51.2 (17.9)	43.8 (17.3)	0.000 (Spain, England) 0.000 (Japan, England) 0.119 (Japan, Spain)
Gender (%)							
Female	230 (42.4)	1926 (56.7)	0.000	511 (49.8)	591 (59.3)	525 (52.5)	0.000
Male	313 (57.6)	1469 (43.3)		515 (50.2)	405 (40.7)	475 (47.5)	
Experience of serious illness (self)^b^ (%)							
Yes	80 (14.7)	1076 (31.7)	0.000	192 (18.7)	330 (33.1)	144 (14.4)	0.000
No^a^	463 (85.3)	2319 (68.3)		834 (81.3)	666 (66.9)	856 (85.6)	
Experience of serious illness (family)^c^ (%)							
Yes	188 (34.6)	2156 (63.5)	0.000	377 (36.7)	692 (69.5)	633 (63.3)	0.000
No^a^	355 (65.4)	1239 (36.5)		649 (63.3)	304 (30.5)	367 (36.7)	
Experience of serious illness (other)^d^ (%)							
Yes	178 (32.8)	547 (16.1)	0.000	130 (12.7)	416 (41.8)	347 (34.7)	0.000
No^a^	365 (67.2)	2848 (83.9)		896 (87.3)	580 (58.2)	653 (65.3)	

*SD* standard deviation

^a^This category includes missing values

^b^Respondents who had experienced serious illness in themselves

^c^Respondents who had experienced serious illness in a family member

^d^Respondents who had taken care of others with a serious illness

The most frequently self-reported profile using both versions of the EQ-5D was full health. The proportion of respondents reporting full health was highest in Japan for both versions of the EQ-5D, and the differences between other countries were statistically significant (*P* < 0.05). After adjusting for differences in age and gender in the EQ-5D-5L samples, the proportion of those reporting full health was still highest among respondents in Japan (66.5%), followed by Spain (54.9%) and England (53.8%). The reduction in the ceiling effect using the EQ-5D-5L compared with the EQ-5D-3L was similar in the UK/England (from 56.9% to 47.6% for the EQ-5D-3L and EQ-5D-5L, respectively) and Japan (from 77.2% to 66.5%).

Analysis of self-reported EQ-5D profile data showed that Japanese respondents employed a much smaller number of health profiles than respondents in other countries. Among the EQ-5D-3L datasets, three health states accounted for 90.1% of Japanese respondents compared with 12 health states in the UK (90.6%). The difference is even more marked for the EQ-5D-5L, where only four health states accounted for 91.4% of Japanese respondents compared with 80 health states in England (90.1%) and 16 health states in Spain (90.0%). Full EQ-5D profile distributions by country and EQ-5D version are available upon request from the authors.

**Fig. 1:**
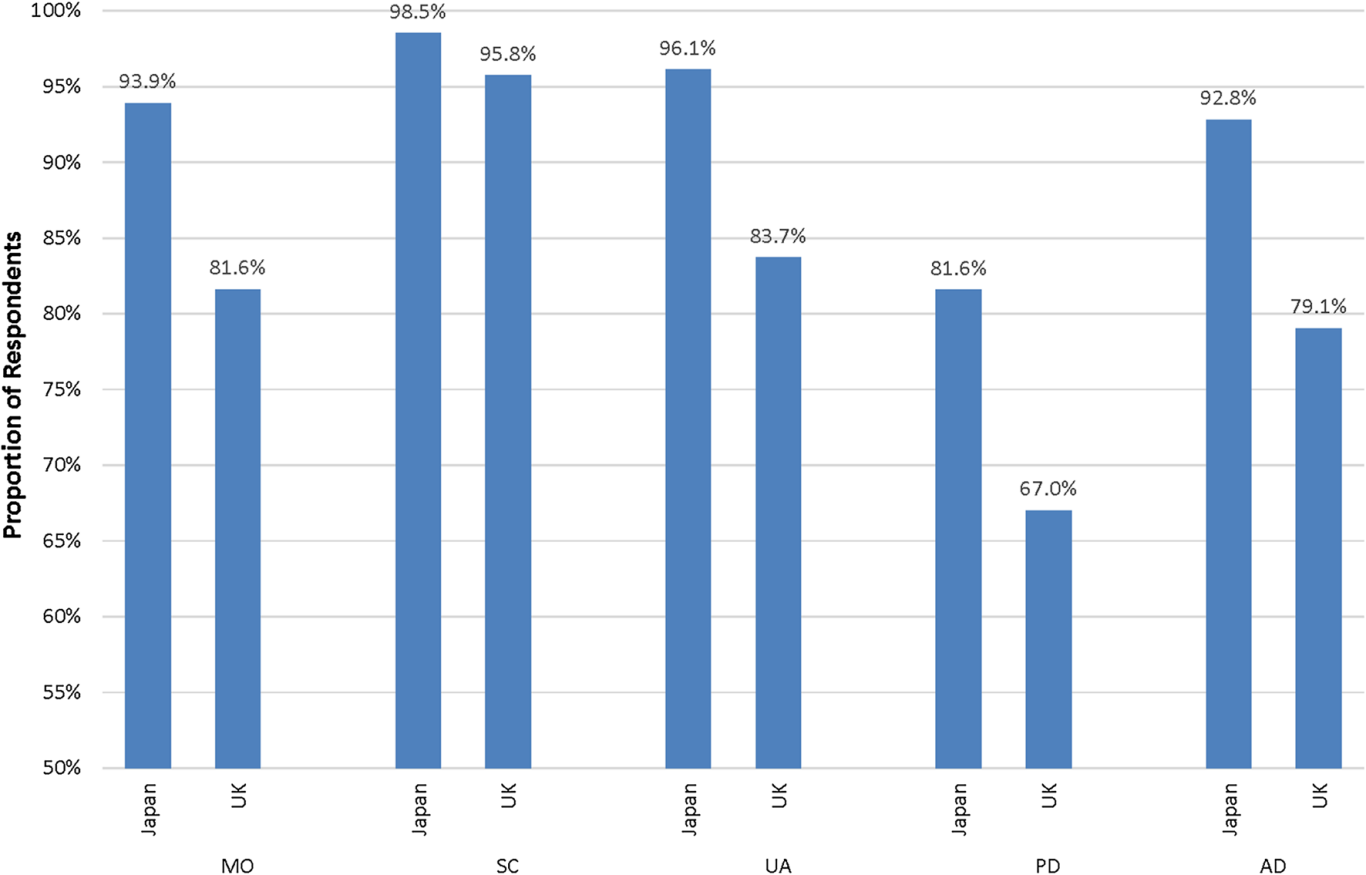
The proportion of respondents in Japan and the UK who reported level one (no problems) by EQ-5D-3L dimension. *MO* mobility, *SC* self-care, *UA* usual activities, *PD* pain/discomfort, *AD* anxiety/depression

**Fig. 2:**
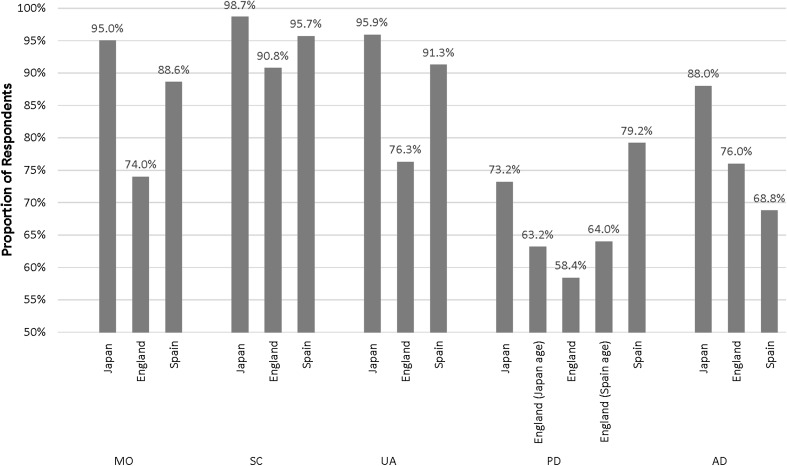
The proportion of respondents in Japan, England, and Spain reporting level one (no problems) by EQ-5D-5L dimension. *MO* mobility, *SC* self-care, *UA* usual activities, *PD* pain/discomfort, *AD* anxiety/depression. Data on the pain/discomfort dimension for England include those adjusted by age distribution of Japan (Japan age) and Spain (Spain age)

Figure 1 shows the proportion of respondents reporting level one for the EQ-5D-3L by dimension and country. In the pain/discomfort dimension, 81.6% of respondents in Japan self-reported level one, compared with 67.0% of respondents in the UK. Figure 2 shows that more respondents in Japan self-reported level one for the EQ-5D-5L than in England and Spain in four dimensions. In the pain/discomfort dimension, a higher proportion of respondents in Spain (79.2%) self-reported level one than in Japan (73.2%) and England (58.4%), although after adjusting for differences in age and gender between the samples, the proportion of respondents reporting level 1 on the pain/discomfort dimension in England was approximately 64%.

The results of modeling respondents’ EQ-VAS scores as a function of their self-reported EQ-5D profiles are shown in Table 3. EQ-VAS scores decreased when the severity of problems increased in any of the five dimensions, and this finding was consistent across countries and EQ-5D versions. Using EQ-5D-3L, pain/discomfort was the most important dimension in explaining respondents’ self-reported EQ-VAS scores in Japan (on average, a one level increase in the pain/discomfort dimension led to a decrease of 11.03 points on the EQ-VAS), whereas in the UK, it was the dimension of usual activities. Using EQ-5D-5L data, anxiety/depression was the most important dimension in explaining differences in EQ-VAS scores in England and Spain; however, in Japan, the most important dimension was usual activities. The adjusted R-squared and results from residual analyses for each model are reported in Table 3. The results from residual analyses suggest no evidence of multicollinearity in the five models. However, there is evidence of residuals with non-normal distributions, heteroscedasticity, and non-linear functional form for some specifications.

**Table 3:** Modeling self-reported EQ-VAS scores by country and EQ-5D version

	Japan EQ-5D-3L		UK EQ-5D-3L		Japan EQ-5D-5L		England EQ-5D-5L		Spain EQ-5D-5L	
	Coef	*P* value	Coef	*P* value	Coef	*P* value	Coef	*P* value	Coef	*P* value
Mobility	−0.88	0.785	−5.87	0.000	−2.87	0.029	−3.46	0.000	−0.35	0.719
Self-care	−5.42	0.394	−6.82	0.000	−4.41	0.128	−0.21	0.836	−3.29	0.065
Usual activities	−9.50	0.018	−8.54	0.000	−9.61	0.000	−4.87	0.000	−5.86	0.000
Pain/discomfort	−11.03	0.000	−6.80	0.000	−4.53	0.000	−3.28	0.000	−4.59	0.000
Anxiety/depression	−7.22	0.003	−8.02	0.000	−5.83	0.000	−6.96	0.000	−7.87	0.000
Constant	115.80	0.000	125.80	0.000	114.94	0.000	105.99	0.000	109.82	0.000
Adjusted *R*-squared^a^	0.2027		0.4226		0.2285		0.5031		0.4026	
*N*	543		3372		1026		996		1000	

Note: (1) Shapiro–Wilk test rejected the null hypothesis of normally distributed residuals in all five regressions at the 5% significance level. (2) The Breusch–Pagan test accepted the null hypothesis that the variance of the residuals is homogeneous at the 5% significance level for the Japan EQ-5D-3L model, but rejected the null hypothesis in all other four models. (3) The regression equation specification error test (RESET) rejected the null hypothesis of linear functional form for the specifications in Japan EQ-5D-5L model and Spain EQ-5D-5L model at the 5% significance level. (4) None of the five models indicated issues with multicollinearity (mean variance inflation factor < 3)

^a^Adjusted *R*-squared is computed using the formula 1 − [(1 − Rsq) × (*N* − 1)/(*N* − *k* − 1)], where Rsq represents *R*-squared (the proportion of variance in the dependent variable that can be explained by the independent variables); *N* represents the number of observations; and *k* represents the number of independent variables

Table 4 reports the mean EQ-5D-3L TTO values for the 17 hypothetical health states valued by respondents in the UK and Japan. In Japan, none of the mean TTO values for the 17 health states were below zero (i.e., none of them were considered as being worse than dead). By contrast, six of the 17 health states in the UK had negative mean TTO values. TTO values for the five mildest health states (11112, 11121, 11211, 12111, 21111) were lower in Japan than in the UK, with the differences being statistically significant at the 5% significance level. However, Japanese TTO values for the remaining 12 more severe health states were all higher than UK values for those health states. The differences were statistically significant at the 5% significance level for 11 health states, but not for health state 22222.

**Table 4:** Comparing the mean TTO values for the 17 hypothetical EQ-5D-3L health states between respondents in Japan and the UK

Health state	Japan	UK	*t* statistics
11112	0.79	0.83	−2.90*
11121	0.79	0.85	−5.13*
11211	0.82	0.87	−4.73*
12111	0.81	0.83	−1.90*
21111	0.78	0.88	−8.05*
11113	0.71	0.39	12.36*
11131	0.64	0.20	15.81*
11312	0.64	0.55	4.01*
11133	0.54	−0.05	20.37*
13311	0.60	0.35	9.74*
32211	0.33	0.15	5.90*
22222	0.51	0.50	0.41
23232	0.41	−0.09	17.54*
32223	0.22	−0.17	13.86*
32313	0.21	−0.15	12.83*
33323	0.09	−0.39	18.81*
33333	0.01	−0.54	29.15*

*Significant at the 5% significance level

Table 5 reports the mean EQ-5D-5L TTO values for the 86 hypothetical health states valued by respondents in Japan and England. For respondents in both countries, only the worst state 55555 was assigned a mean TTO value below zero. Mean TTO values were higher in Japanese respondents for 63 of the 86 hypothetical health states, with 19 of those differences being statistically significant (*P* < 0.05). No clear pattern was observed between the severity of health states and the presence of higher values from Japanese respondents; the 19 health states included mild states (e.g., 11112) and severe states (e.g., 55555). In only one case was a statistically significant higher value observed for English raters (state 35332).

**Table 5:** Comparing the mean TTO values for the 86 hypothetical EQ-5D-5L health states between respondents in Japan and England

Health state	Japan	England	*t* statistics	Health state	Japan	England	*t* statistics
11112	0.91	0.85	2.41*	31524	0.46	0.45	0.18
11121	0.90	0.89	0.71	31525	0.39	0.43	−0.58
11122	0.86	0.79	2.15*	32314	0.52	0.51	0.23
11211	0.91	0.89	0.83	32443	0.45	0.29	2.48*
11212	0.81	0.82	-0.23	33253	0.41	0.40	0.04
11221	0.85	0.84	0.13	34155	0.36	0.24	1.70
11235	0.60	0.53	1.24	34232	0.53	0.55	−0.45
11414	0.60	0.41	3.23*	34244	0.34	0.26	1.23
11421	0.72	0.65	1.49	34515	0.30	0.32	−0.26
11425	0.54	0.53	0.09	35143	0.40	0.27	2.02*
12111	0.89	0.87	1.26	35245	0.30	0.18	1.89
12112	0.82	0.81	0.13	35311	0.60	0.51	1.60
12121	0.87	0.81	1.72	35332	0.38	0.59	−3.28*
12244	0.50	0.32	2.73*	42115	0.48	0.41	1.14
12334	0.61	0.44	2.55*	42321	0.59	0.54	0.89
12344	0.51	0.25	3.63*	43315	0.39	0.42	−0.37
12513	0.61	0.61	−0.01	43514	0.31	0.36	−0.70
12514	0.53	0.44	1.51	43542	0.33	0.23	1.50
12543	0.47	0.32	2.22*	43555	0.13	0.06	1.05
13122	0.75	0.81	−1.34	44125	0.38	0.32	0.78
13224	0.61	0.49	2.14*	44345	0.23	0.21	0.17
13313	0.63	0.69	−1.13	44553	0.17	0.09	1.06
14113	0.70	0.69	0.31	45133	0.49	0.36	1.85
14554	0.31	0.15	2.18*	45144	0.32	0.17	2.39*
15151	0.54	0.42	2.08*	45233	0.36	0.33	0.43
21111	0.90	0.89	0.37	45413	0.32	0.34	−0.32
21112	0.81	0.83	−0.39	51152	0.36	0.35	0.19
21315	0.60	0.54	1.02	51451	0.33	0.26	1.08
21334	0.54	0.50	0.50	52215	0.40	0.35	0.78
21345	0.42	0.43	−0.18	52335	0.32	0.33	−0.09
21444	0.40	0.15	3.93*	52431	0.43	0.54	−1.79
22434	0.45	0.53	−1.24	52455	0.15	0.07	1.07
23152	0.49	0.39	1.42	53221	0.58	0.58	−0.03
23242	0.52	0.44	1.30	53243	0.36	0.23	1.94
23514	0.54	0.40	2.29*	53244	0.26	0.12	2.00*
24342	0.46	0.36	1.48	53412	0.36	0.44	−1.23
24443	0.38	0.33	0.80	54153	0.28	0.27	0.22
24445	0.30	0.16	2.13*	54231	0.40	0.40	−0.05
24553	0.22	0.33	−1.49	54342	0.34	0.18	2.17*
25122	0.55	0.52	0.40	55225	0.21	0.17	0.52
25222	0.57	0.59	−0.54	55233	0.27	0.28	−0.06
25331	0.56	0.53	0.47	55424	0.17	0.25	−1.05
31514	0.45	0.39	0.98	55555	−0.02	−0.08	2.69*

*Significant at the 5% significance level

Three pairs of health states in the EQ-5D-3L valuation data in the UK and Japan differed only on the pain/discomfort dimension. Analysis of those states showed that Japanese respondents traded off less time to avoid problems in the pain/discomfort dimension than UK respondents. The biggest between-country difference in mean TTO values was reported between health states 11121 and 11131, which would represent a decrease of 0.65 in TTO values in the UK compared with 0.15 in Japan.

Seven pairs of health states in the EQ-5D-5L in the English and Japanese valuation studies differed only in the pain/discomfort dimension. Specifically, four pairs differed between level 1 (no problem) and level 2 (mild problem), two pairs differed between level 1 (no problem) and level 4 (severe problem), while one pair differed between level 3 (moderate problem) and level 4 (severe problem). Comparing the mean TTO values between respondents in the two countries showed that Japanese respondents traded off either similar or less time to avoid problems in pain/discomfort than English respondents. The biggest difference in mean TTO values was reported between health states 12334 and 12344, which would represent a decrease of 0.19 in TTO values in England compared with 0.10 in Japan.

Regression analysis of the linked self-reported and valuation datasets showed that respondents’ self-reported pain/discomfort was not significant in explaining the TTO values in Japan for EQ-5D-3L (*P* = 0.395) and EQ-5D-5L (*P* = 0.299), nor the UK for EQ-5D-3L (*P* = 0.159). However, it has significant positive effect in explaining the EQ-5D-5L TTO values in England (*P* < 0.05).

## Discussion

This is the first study to carry out an in-depth examination of the comparability of EQ-5D self-rated health status and valuation data from Japanese and European respondents, with a particular focus on pain/discomfort. A number of findings were clear from the empirical analyses in this study.

First, respondents in Japan tend to report better health in general than respondents in England/UK and Spain. Second, with respect to pain/discomfort, respondents in Japan reported problems less frequently than respondents in England/UK, but slightly more frequently than respondents in Spain. Third, Japanese respondents used a much smaller number of health states to describe their health than respondents in either of the other two countries, and Spanish respondents also used substantially fewer health states than respondents in England. Fourth, in the EQ-5D-3L valuation study, respondents in Japan were *more* willing to trade off time for the mildest health states, but *less* willing to trade off time for the severe health states compared with respondents in the UK. For nearly three-fourths of the EQ-5D-5L health states for which values were obtained, Japanese respondents’ values were higher than those from English respondents. However, in contrast with EQ-5D-3L values, there was no clear pattern between this and the severity of the states. Fifth, in the EQ-5D-3L and EQ-5D-5L valuation studies, Japanese respondents were willing to trade off less time to avoid problems in the pain/discomfort dimension than respondents in England/UK. However, the differences in TTO values between respondents in Japan and England are much smaller than in the EQ-5D-3L valuation study.

It is not clear where these differences stem from, though similar findings have been reported previously. For example, in a comparison of EQ-5D results from 20 countries in a diabetes clinical trial, researchers found substantial variation in the reporting of functional health problems, but noted that the variation could not be explained by differences in demographic variables, clinical risk factors, or rates of complications [22]. They suggested that the unexplained variability meant there were important problems of comparability across settings.

One possible cause of the differences found here is that the way terms used to describe health, e.g., the severity labels, varies across countries. For example, Luo et al. found that the interpretation and use of EQ-5D-5L response labels (e.g., ‘slight’, ‘moderate’, and ‘severe’) varied across Chinese, Malay, and English speakers in Singapore [23], whereas the English version gave similar outcomes in Chinese and non-Chinese English speakers in the same country [24], suggesting that there was no effect of culture on responses. Although a strict protocol is followed in producing other language versions of EQ-5D [25], it may not always be possible to find identical terms in all languages. There is also evidence suggesting that Japanese respondents might be less willing to report pain than those in Europe, possibly due to a tendency within the Japanese culture for pain to be repressed and controlled rather than shared or expressed [26].

Our findings on the reporting of pain/discomfort coincide with those of earlier multi-country studies that showed a tendency towards lower rates or intensity of self-reported pain in Japan than in other countries [7, 8, 27]. Despite lower rates of self-reported pain/discomfort in Japan, we found that this was the most important dimension in explaining respondents’ self-reported EQ-5D-3L VAS scores. There are two possible explanations for this discrepancy. First, while the EQ-VAS scores and self-reported EQ-5D profiles measure how good or bad respondents rate their currently experienced health status, the TTO valuation task evaluates health states that are hypothetical to the respondents. Second, the tasks involved EQ-VAS scores rating and TTO valuation, which are individually very different. It is possible for a respondent to rate a health state as poor on the EQ-VAS, but still not be willing to trade off any life years to avoid it (e.g., because of religious beliefs about the sanctity of life, being the primary caregiver to a small child, or having a very low personal discount rate). As the EQ-5D profile variables are treated as continuous, the importance of each EQ-5D dimension in explaining the EQ-VAS reflects the average effect in a dimension between two neighboring levels. An alternative approach is to treat the EQ-5D profile variables as dummies (i.e., one for each level and dimension). However, this would leave some categories with rather small sample sizes, particularly for severe levels (*n* < 5). Those results from the EQ-5D-3L data were not confirmed using EQ-5D-5L data. Given the design of the current study, it was not possible to determine whether the difference in findings was due to changes in methods or perceptions of the importance of pain/discomfort over time. It should also be noted that the lowest rates of pain/discomfort were observed in Spain. Other studies have also reported relatively low rates of self-reported problems on the EQ-5D descriptive system in the general population in Spain compared with other European countries although not on the EQ-VAS [10]. Similar findings have been reported for Spain using other instruments, such as the Brief Pain Inventory [28].

The comparison of valuation data also showed differences between countries. The Japanese EQ-5D-3L valuation data showed a tendency to compress towards the middle of the scale. A mid-range response style and lower levels of extreme response style have been reported in some studies in Japanese subjects [29–31], though it is not clear whether such an effect may also be present in valuation studies. Furthermore, we found that respondents in Japan were less willing to trade off time to avoid pain/discomfort on the EQ-5D-3L than respondents in the UK. It should be noted that the 17 health states valued by the Japanese respondents were a subset of the health states in the UK valuation study. The TTO values may be influenced by the mix of severity in the set of states presented. This may affect observed differences in TTO values.

This compression of values in Japan relative to UK values was no longer observed when analyzing results from the EQ-5D-5L. Only the worst state (i.e., 55555) was rated worse than death in both countries. Almost three-quarters of the EQ-5D-5L health states were given higher values by Japanese respondents compared with English respondents, indicating more reluctance to trade off time among Japanese respondents. However, unlike with the EQ-5D-3L data, these higher ratings were spread across all levels of severity. It is possible that these differences are due to changes in the methods that were used in the TTO valuation tasks between the two versions of EQ-5D, or changes in perceptions of health states, and/or relative importance assigned to different dimensions over time. However, it is not possible to answer it definitively here.

### Limitations

Ideally, samples from Japan and European countries used to explore differences in the self-reporting and valuation of pain would have identical distributions for all factors that might influence results. However, it was not possible to control for all relevant variables, although we controlled for the effects of age and gender. It remains unclear whether differences in the rates of health problems between countries and other unobserved characteristics may have led to the differences we observed.

Furthermore, the EQ-5D-3L data used in this study were collected in the 1990s and may no longer be applicable to the present populations in the UK and Japan; however, we do not consider this to be a limitation of the present analysis as we were interested in comparing results between countries and not in exploring whether the data collected then would be relevant today. The fact that it was possible to compare findings from two different variants of the instrument at two different time points could in fact be considered a strength of the study because it gives an indication of the robustness of the results.

The possible misspecifications for modeling EQ-VAS scores by the EQ-5D dimensions should be noted. Although the assumption of normality does not hold in the five models and, as a consequence, will have an impact on the *P* values, the estimated coefficients themselves will still be consistent. Four models reported heteroscedasticity in the residuals and two models reported non-linear functional forms. These misspecifications might be explained by variables that are not included in modeling the EQ-VAS, but have an impact on the EQ-VAS, such as other health dimension(s) that are not covered by EQ-5D. If those variables are correlated with the EQ-5D dimensions, our estimated coefficients could be biased. Similar issues have been observed in previous studies [18, 32, 33].

Finally, only data from two European countries were available for the present study and it is not clear whether results can be extrapolated to respondents in other Western countries.

### Implications

Our findings have a number of implications. First, care should be taken when comparing and aggregating clinical data on pain between different countries, because respondents may use different criteria when responding, which could potentially lead to the same treatment being more or less effective in different countries. Second, the differences between respondents in Japan and European countries in self-reported and valuation behaviors could have a substantial effect on the results of cost–utility analyses. For instance, while applying the EQ-5D-3L instrument, the compression of values on the utility scale and the better baseline pain scores observed in Japan may result in relatively small improvements with treatment. Third, what constitutes a minimally important difference for EQ-5D index may be different between Japan and other countries. Fourth, if the findings related to pain/discomfort also applied to other pain measures used as inclusion criteria for clinical trials, then they might lead to questions about whether identical inclusion criteria for clinical trials are in fact being used across countries.

## Conclusions

This study provides prima facie evidence of differences between Japan, UK/England, and Spain in the self-reporting and valuation of health, including pain/discomfort, when using EQ-5D in general population samples. The findings suggest the need for caution when comparing and/or aggregating EQ-5D data across the countries. Specifically designed studies, including the use of qualitative research and vignette techniques [34], would be helpful in exploring these issues further and confirming the findings.
